# Ethical Challenges and Strategies in Nursing Doctoral Supervision: A Systematic Mixed‐Method Review

**DOI:** 10.1111/jan.70298

**Published:** 2025-10-23

**Authors:** Tove Godskesen, Maria Grandahl, Amalie Nilsen Hagen, Stefan Eriksson

**Affiliations:** ^1^ Faculty of Nursing and Health Sciences Nord University Bodø Norway; ^2^ Centre for Research Ethics & Bioethics, Department of Public Health and Caring Sciences Uppsala University Uppsala Sweden; ^3^ Department of Women's and Children's Health Uppsala University Uppsala Sweden

**Keywords:** doctoral education, ethical challenges, health sciences, nursing sciences, strategies, systematic review

## Abstract

**Aim:**

To identify and address ethical challenges in doctoral supervision within nursing and health sciences and propose strategies to overcome them.

**Design:**

Following PRISMA guidelines, this mixed‐method systematic review synthesises findings from quantitative, qualitative and mixed‐methods studies published in English between 2014 and 2025. Studies were included if they examined ethical challenges in doctoral supervision and strategies to address them within nursing and health sciences. Exclusion criteria encompassed reviews, books, editorials, opinion papers, conference papers, studies unrelated to nursing or health sciences or published before 2014.

**Data Sources:**

A systematic search was conducted in CINAHL, Education Source, ERIC, PubMed, Scopus and Web of Science Core Collection, yielding 1100 citations.

**Review Methods:**

The methodological quality of included studies was assessed using the STROBE checklist for quantitative studies and the COREQ framework for qualitative studies. The findings were then synthesised and thematically organised.

**Results:**

Eleven studies met the inclusion criteria: four quantitative, four qualitative and three mixed methods. Ethical challenges in doctoral supervision emerged at three levels: individual (e.g., misaligned expectations, inadequate feedback, student adjustment difficulties), institutional (e.g., high student–supervisor ratios, limited support structures), and cultural (e.g., differing norms around autonomy and academic authority). Supervisors also reported role conflicts. Strategies to address these challenges included improved communication, supervision agreements, institutional support and targeted training.

**Conclusions:**

Ethical challenges in supervision are shaped by individual, institutional and cultural factors. Addressing them requires multi‐level strategies, including clear expectations, feedback mechanisms, structured training and culturally sensitive supervision practices. Applying ethical principles fosters a transparent and supportive academic environment that enhances doctoral outcomes.

**Implications for the Institutions:**

Universities should adopt multi‐level strategies, including supervisor training, mentorship structures and culturally informed policies, to strengthen the ethical integrity and effectiveness of doctoral supervision.

**Impact:**

*What problem did the study address?*: This study synthesises ethical challenges in doctoral supervision within nursing and health sciences, focusing on communication barriers, institutional constraints and the transition from clinical practice to academia. *What were the main Findings?*: Misaligned expectations between supervisors and students, inadequate feedback and structural limitations, negatively impact the quality of supervision. Doctoral students struggle to adapt to academic expectations, while supervisors face challenges in balancing multiple roles. Effective communication, institutional support and targeted training programmes are essential for improving supervisory experience. *Where and on whom will the research have an impact?*: The research will inform universities and institutions offering doctoral education in nursing and health sciences. It will benefit doctoral students, supervisors and academic administrators by providing insights and strategies to enhance supervision quality and promote ethical practices.

**Reporting Method:**

This systematic review follows the Preferred Reporting Items for Systematic Reviews and Meta‐Analyses (PRISMA) guidelines.

**Patient or Public Contribution:**

No patient or public involvement.

**Summary**

- What does this paper contribute to the wider global clinical community?
  ○ Provides critical insights into ethical challenges in doctoral supervision within nursing and health sciences, highlighting the need for clear expectations and effective communication.
  ○ Emphasises the importance of institutional support, including mentorship programmes and supervisor training.
  ○ Advocates for the integration of ethical principles into supervisory practices to foster a fair, transparent and supportive academic environment.

## 1 Introduction

As healthcare evolves through technological advancements and an increasing emphasis on evidence‐based practice, developing the next generation of nurse researchers has become increasingly important (Breslin et al. 2015). Doctoral nursing education is increasingly acknowledged as a key driver in advancing clinical practice and research capabilities, enabling nurses to address the complexities of modern healthcare (Joseph et al. 2021). The World Health Organization (WHO) has underscored the importance of a well‐educated nursing workforce as essential to tackling global health challenges (WHO 2021). Doctoral programmes are crucial in preparing researchers to address complex healthcare challenges and create knowledge that informs clinical practice (Guarimata‐Salinas et al. 2024). However, while essential for the future of nursing, doctoral education also presents significant challenges, particularly for supervision and ethical responsibilities (Peters et al. 2023). These challenges are not limited to any region or educational system. Rather, supervision in doctoral nursing education is a global concern, shaped by diverse institutional structures, cultural norms and professional standards (Dobrowolska et al. 2021). In the Nordic countries, doctoral programmes in nursing and caring sciences exhibit notable variations in their organisation and requirements. For example, Norway and Denmark typically require three years of full‐time study, whereas Sweden and Finland extend this to four years (Slettebø et al. 2013). In the United States and the United Kingdom, there are two main types of nursing doctorates, the Doctor of Nursing Practice (DNP) and the Doctor of Philosophy (PhD). The PhD is research‐focused, aimed at advancing nursing knowledge through scholarly inquiry, while the DNP is a professional doctorate designed to prepare clinical leaders who apply research to practice. In the United States, the DNP is a required entry‐level degree for advanced nursing practice (ANP), ensuring that nurses are equipped to lead evidence‐based care (Dobrowolska et al. 2021). These structural variations reflect broader cultural and institutional factors that shape the doctoral experience, influencing both student progression and the supervisory process. Yet, despite these differences, one aspect remains unchanged: the pivotal role of supervision in shaping doctoral students' academic and professional development (van Rooij et al. 2021).

Supervision in nursing doctoral education goes beyond academic guidance as it carries significant ethical implications that influence professional relationships, power dynamics and research integrity (Löfström and Pyhältö 2020). The applied nature of nursing and health sciences research further complicates these ethical dimensions, as close collaboration between academia and clinical practice imposes moral and professional responsibilities for both students and supervisors (Thompson et al. 2005; Löfström et al. 2023). Supervisors serve as role models who impart ethical values, influencing students' approaches to research, clinical practice and professional conduct (Pizzolato and Dierickx 2022). Establishing supervisory relationships grounded in fairness, transparency and mutual respect is essential to fostering a research culture that upholds ethical integrity (Darder Mesquida and Pérez Garcias 2015; Jackson et al. 2021a, 2021b).

Doctoral students in nursing often face unique challenges during their transition from clinical practice to academia (Dobrowolska et al. 2021). While they bring extensive practical experience, many struggle to adapt to academic expectations, including theoretical frameworks, research methodologies and scholarly writing (Severinsson 2015; Louise McIntosh 2023). The shift from a fast‐paced clinical environment to the demands of academic research can be overwhelming, making effective and ethical supervision crucial for student retention and success (Scheese et al. 2023; Murray et al. 2014). Additionally, imbalances in supervisory relationships, unclear authorship agreements and insufficient institutional support can lead to ethical dilemmas such as conflicts of interest when a supervisor priorities their own research agenda over the student's academic development (Kondakci et al. 2024; Löfström and Pyhältö 2017).

Given these challenges, examining the ethical dimensions of supervision in nursing doctoral education is both timely and necessary. Despite growing attention to doctoral supervision in general (Löfström and Pyhältö 2020, 2017), there has been, to our knowledge, no systematic analysis of the ethical dimensions specific to supervision in nursing doctoral programs, where the applied nature of research and close clinical–academic relationships present unique ethical challenges (Haley et al. 2025). Institutions have a fundamental ethical responsibility to provide an environment that promotes justice, ensures student well‐being (beneficence), and prevents harm (non‐maleficence) (Volkert et al. 2018). Addressing these concerns is necessary for improving doctoral education and for shaping the next generation of nurse researchers who will advance healthcare both in clinical practice and research (Schmidt and Hansson 2018).

## 2 The Review

### 2.1 Aims

To identify and address ethical challenges in doctoral supervision within nursing and health sciences and to propose strategies to overcome them. Specifically, this review addresses two research questions:

1. What factors contribute to ethical challenges in doctoral supervision within nursing and health sciences?
2. What strategies are suggested to address these ethical challenges?

### 2.2 Design

This study employs a systematic mixed methods review approach (Pluye and Hong 2014; Aromataris et al. 2024) adhering to Preferred Items for Systematic Reviews and Meta‐Analysis (PRISMA) guidelines (Page et al. 2021). This design integrates qualitative and quantitative findings to provide a nuanced understanding of ethical challenges in doctoral supervision.

### 2.3 Search Methods

On December 6, 2024, a medical librarian at XX University conducted a systematic search across multiple databases, including CINAHL, Education Source, ERIC, PubMed, Scopus and the Web of Science Core Collection. On September 15, 2025, an updated search was conducted. The search terms and combinations were formulated based on the specific research questions. To enhance the rigor and relevance of the search strategy, we collaborated with a medical librarian. Keywords were developed using Medical Subject Headings (MeSH), but the search terms were modified to accommodate the terminology specific to each database. The results were validated by another medical librarian to confirm the accuracy of the search.

The finalised search strategy included the following key terms: “Ethics” [MeSH] OR “Moral” [MeSH] OR “Value” OR “Research integrity”, AND “Supervis*” OR “mentorship” OR “advisor*” OR “academic advising”, AND “Doctoral degree*” OR “doctoral program*” OR “doctoral nursing program*” OR “doctoral stud*” OR “PhD”, AND “Nurses”[Mesh] OR “nurs”* OR “health care science” OR “health science*” OR “caring science*” OR “nursing science*” OR “caring research” OR “nursing research”. These terms were combined using Boolean operators to ensure a comprehensive search that captured the relevant literature across the selected databases. To avoid constraining the conceptual scope of the review, we applied a broad search strategy using general ethical terms rather than pre‐defining specific issues such as power dynamics. This approach aligns with recommendations for qualitative evidence synthesis, which caution against overly narrow search terms that may exclude relevant studies using different conceptual framings (Booth et al. 2021).

Recognising that variations in indexing and terminology exist across different databases, the search terms were tailored for each database to optimise retrieval. For instance, while some databases require the use of specific filters or tags to refine results, others allowed for more flexible keyword combinations. The tailored search strategy is provided in Appendix S2.

The screening process consisted of several steps: removal of duplicate articles; screening remaining articles based on the relevance of their titles and abstracts; full‐text screening of remaining articles; finally, we proceeded to extract data. The screening was performed by two reviewers, TG and SE. In instances where discrepancies arose regarding the inclusion or exclusion of specific articles, these were resolved through discussion and consensus.

All authors hold doctoral degrees and bring relevant expertise to the study. TG is Professor of Nursing and Associate Professor of Medical Ethics, with extensive experience in systematic reviews and nursing ethics. MG, Associate Professor of Paediatric Caring Science, has broad experience in both quantitative and qualitative methods. ANH, Associate Professor in Nursing, specialises in quantitative research. SE, Associate Professor of Research Ethics, has extensive experience in research ethical issues. All authors also have experience in supervising PhD students. This combined expertise ensured a rigorous and reflective quality appraisal process.

### 2.4 Inclusion and Exclusion Criteria

This review included peer‐reviewed empirical studies—qualitative, quantitative and mixed method studies—published between January 1, 2014, and September 15, 2025, and indexed in Scopus and Web of Science. Study selection was guided by the PICO framework (see Table 1). This approach aligns with the *Journal of Advanced Nursing's* (JAN) editorial guidelines, which emphasise the use of high‐quality, peer‐reviewed literature from reputable academic databases. JAN prioritises the inclusion of current, evidence‐based sources to uphold scholarly rigour, clinical relevance and the advancement of nursing science. Exclusion criteria comprised literature reviews, grey literature (e.g., dissertations, reports or non‐peer‐reviewed materials), studies unrelated to nursing or health sciences and publications dated before 2014.

**TABLE 1:** The PICO framework.

PICO criteria	
P (Population)	Doctoral students in nursing and health sciences
I (Intervention)	Identification and analysis of ethical challenges in doctoral supervision
C (Comparison)	Not applicable
O (Outcome)	Strategies to address and mitigate ethical challenges in doctoral supervision

### 2.5 Search Outcome

**FIGURE 1:**
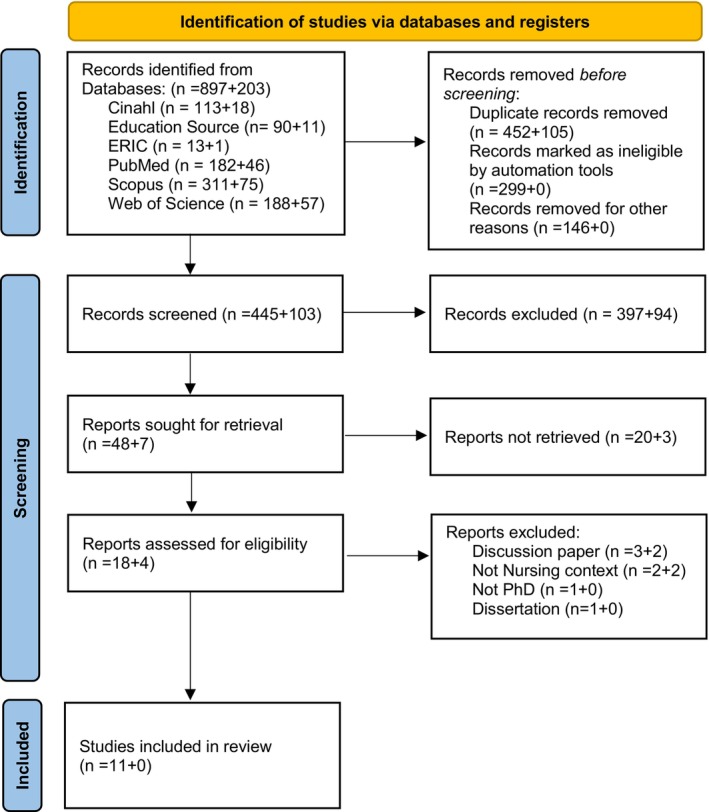
PRISMA 2020 flow diagram for new systematic reviews which included searches of databases and registers only. *Source:* Page MJ, et al. BMJ 2021;372:n71. doi: 10.1136/bmj.n71. This work is licensed under CC BY 4.0. To view a copy of this license, visit https://creativecommons.org/licenses/by/4.0/ .

The database searches identified 897 studies (initial search) + 203 (updated search), of which 452 + 105 were duplicates and therefore removed in Rayyan (Ouzzani et al. 2016). The remaining 445 + 103 studies were screened by title and abstract. Of these, 48 + 7 were included for full‐text review based on eligibility criteria. Twenty +3 studies were excluded after full‐text review, leaving 18 + 4 for assessment. Of these, 7 + 4 were excluded for various reasons, resulting in 11 studies being included in this systematic review. The study selection process is outlined in Figure 1 and Appendix S1.

### 2.6 Quality Appraisal

The methodological quality of the included studies was systematically evaluated using established checklists, detailed in Appendix S3. Quantitative studies were assessed using the STROBE (Strengthening the Reporting of Observational Studies in Epidemiology) checklist (von Elm et al. 2014), while qualitative studies were evaluated using the COREQ (Consolidated Criteria for Reporting Qualitative Research) framework (Tong et al. 2007). For mixed‐methods studies, the quantitative component was appraised using STROBE and the qualitative component using COREQ, ensuring a rigorous assessment of both methodological approaches.

To enhance rigourrigor and validity, MG, ANH and TG held an initial calibration meeting to ensure a shared understanding of the quality appraisal criteria. Two researchers (MA and ANH) then independently assessed the quality of the included studies. They met regularly to compare evaluations, discuss discrepancies and reach consensus. When disagreements remained, a third researcher (TG) was consulted to facilitate resolution. This structured, collaborative approach helped minimise bias and ensured consistency throughout the appraisal process.

None of the included articles fully met STROBE or COREQ criteria. In addition, some criteria in mixed‐methods studies were deemed not applicable (NA), neither for STROBE nor COREQ checklists. However, despite these limitations, the studies were overall considered methodologically adequate and robust, providing valuable data relevant to the aim of this review.

### 2.7 Data Abstraction

A standardised data extraction form was developed to ensure systematic organisation of key study details, including:

– Author, year and country
– Aim of the study
– Design, methods and participants
– Ethical challenges identified
– Strategies for addressing challenges

Two authors (TG and SE) independently extracted data from the included studies, ensuring consistency and reliability. For quantitative studies, findings were summarised using a narrative synthesis approach, including contextual interpretation to understand the prevalence and scope of ethical challenges (Pluye and Hong 2014). Key findings from qualitative studies were recorded to identify recurring patterns and underlying meanings. We used a side‐by‐side comparison approach, examining results from quantitative and qualitative studies together to explore how they matched, diverged or complemented each other. This allowed us to, for example, identify areas where quantitative findings (e.g., frequency of communication problems) were expanded upon by qualitative insights that explored the relational, cultural or institutional dimensions underlying those problems. Regular discussions were held between TG and SE throughout the process to ensure systematic organisation of the data and to accurately capture key insights across both data types.

### 2.8 Data Synthesis

The findings were organised and synthesised using thematic analysis (Braun and Clarke 2006). Guided by the two research questions, the synthesis aimed to explore ethical challenges in doctoral supervision, including their causes, implications and potential solutions. Patterns within the extracted data were categorised into themes, which two authors (TG and SE) independently identified and verified. All authors contributed to refining and finalising the themes.

As this was a mixed‐methods systematic review with a primarily descriptive and interpretive aim, we did not assess statistical power. The focus was on synthesising insights and patterns across studies to explore ethical challenges in doctoral supervision, rather than drawing generalisable conclusions based on inferential statistics.

## 3 Results

### 3.1 Study Characteristics

The included articles were conducted in diverse settings, representing high‐income countries such as the United States (Volkert et al. 2018; Fang and Bednash 2017; Fang et al. 2016; Taylor et al. 2018; Wieck et al. 2014), Finland (Anttila et al. 2024), Denmark (Raffing et al. 2017), Australia (Geraghty and Oliver 2018) and Portugal (Alves et al. 2023). Studies also included insights from lower‐ and middle‐income contexts, such as South Africa (Havenga and Sengane 2018), and regional analyses of East and Southeast Asia (Molassiotis et al. 2020).

A total of 4.221 participants were included across all 11 studies. Sample sizes varied considerably, ranging from qualitative studies with 15–20 participants, e.g., Havenga and Sengane (2018) and Raffing et al. (2017), to large‐scale surveys involving over 900 participants (Fang and Bednash 2017; Fang et al. 2016). The participants comprised a diverse group, including doctoral students and supervisors, programme coordinators and faculty members, representing varied levels of experience and institutional roles. Of the 11 studies, nine articles focused exclusively on nursing and/or health sciences (Volkert et al. 2018; Fang and Bednash 2017; Fang et al. 2016; Wieck et al. 2014; Raffing et al. 2017; Geraghty and Oliver 2018; Alves et al. 2023; Havenga and Sengane 2018; Molassiotis et al. 2020). The remaining two studies adopted an interdisciplinary approach, including health sciences as part of their scope (Taylor et al. 2018; Anttila et al. 2024) (Table 2).

**TABLE 2:** Findings from the included studies.

Author (year), title and country	Aim	Context	Design, methods and participants	Findings: challenges in doctoral supervision	Findings: strategies to overcome the challenges
Alves et al. (2023), *The value of doctoral education in the intersection of the multiple purposes of higher education*. Portugal	To explore the perceived value of doctoral education (DE) within the intersection of higher education's multiple purposes in the European knowledge society. It sought to understand how various stakeholders, including supervisors and doctoral students in social and health sciences, conceptualise the value of DE and its role in addressing higher education's goals.	European doctoral programs in social and health sciences.	*Design*: Qualitative study. *Method*: Focus group interviews. *Participants*: 105 participants. Members of scientific and monitoring committees, supervisors, PhD holders and doctoral candidates.	Misalignment in roles, responsibilities and research outcomes, e.g., emphasis on publications over learning. Pressure to prioritise measurable outputs over knowledge co‐creation, leading to stress and burnout. Unclear communication and inadequate feedback undermine trust and increase stress. Conflicts between institutional demands and personal academic aspirations create ethical tensions. Supervisors often overestimate their support, while students feel under‐supported. Absence of co‐constructional engagement hinders academic progress and causes frustration. Differences in styles create tensions, affecting student satisfaction and progression. When supervisors and students have differing expectations about roles, responsibilities and research outcomes, e.g., focus on measurable outputs such as publications. Emotional and instrumental support gaps leave students feeling unsupported. Infrequent supervision and unmet expectations increase dropout risks, especially in health sciences.	Establishing mutual expectations through open and transparent dialogue at the outset of the supervisory relationship. Cultural sensitivity to foster supportive and inclusive relationships is needed. Implementing mental health resources and peer support networks for doctoral students to mitigate stress and improve well‐being.
Anttila et al. (2024), *Doctoral supervisors' and supervisees' perceptions on supervisory support and frequency of supervision—Do they match?* Finland	To understand the alignment between the doctoral candidates' and their supervisors' perceptions of good supervision and the frequency of supervision. Also, the interrelationship between the frequency of supervision, thesis format, satisfaction with the supervision, drop‐out intention and study progress.	Multi‐field research university in Finland. Multi‐disciplinary (including health sciences).	*Design*: Mixed‐method study. *Methods*: Questionnaire and qualitative analysis of open‐ended questions. *Participants*: 768 doctoral candidates and 561 doctoral supervisors. Doctoral supervisors and supervisees.	Supervisors emphasised doctoral candidates' own responsibility and the reciprocal nature of supervision regarding the informational support, whereas doctoral candidates did not. Supervisors overestimate support, while students feel under‐supported. The supervisors more often reported supervising an individual candidate weekly (43%) compared to the candidates' perceptions (26%). Limited co‐constructional engagement hinders academic progress and causes frustration. Differences in styles create tensions, affecting satisfaction and progression. Emotional and instrumental support gaps leave students feeling unsupported. Infrequent supervision and unmet expectations increase dropout risks, especially in health sciences.	Clear and open discussions about supervision frequency, feedback timelines and mutual roles to minimise misunderstandings. Supervisors should verbalise supervisory activities more, to create a shared understanding. Enhancing co‐constructional and individualised support to meet diverse needs of doctoral candidates. Regular feedback sessions between supervisors and students were highlighted as a way to ensure continuous improvement and address issues proactively. Develop institutional frameworks to support both supervisors and supervisees. These might include mentoring programs, peer support groups and workshops focusing on building effective supervisory relationships. Creating safe spaces for dialogue and fostering a culture of trust and providing practical, instrumental guidance on academic tasks and research challenges.
Fang et al. (2016), *Identifying barriers and facilitators to nurse faculty careers for PhD nursing students*. USA	To identify barriers and facilitators to academic careers for doctoral (PhD) nursing students.	U.S. PhD nursing programs.	*Design*: Quantitative study. *Method*: Cross‐sectional questionnaire. *Participants*: 933 participants. Nursing PhD students.	Transitioning from clinical practice to academia was described as a significant adjustment, with students often feeling unprepared for the demands of teaching and research roles. Many students felt non‐confident in reviewing and writing grant proposals, while more likely to feel confident in developing curricula and teaching general nursing courses and laboratory/clinical courses.	PhD programs should provide their students with adequate research training to prepare them to become research scientists.
Fang and Bednash (2017), *Identifying barriers and facilitators for future nurse faculty careers for DNP students*. USA	To identify barriers and facilitators to academic careers for Doctor of Nursing practice (DNP) students.	U.S DN Practice programs.	*Design*: Quantitative study. *Methods*: Cross‐sectional questionnaire. *Participants*: 853 participants. Nursing DNP students.	DNP students lack confident in conducting independent research, publishing research or writing grant proposals, impacting their academic career prospects. Many feel unprepared or overwhelmed when shifting from clinical practice to teaching roles. Perceived as inadequate, discouraging potential nurse faculty from pursuing academic careers.	DNP programmes must address the need for informatics expertise. Integrate targeted training in teaching competencies, particularly in areas like informatics and classroom instruction. This would better prepare students for the demands of academic roles and build their confidence in teaching.
Geraghty and Oliver (2018), *In the shadow of the ivory tower: Experiences of midwives and nurses undertaking PhDs*. Australia	To gain an understanding of the experiences of nurses and midwives enrolled in a PhD, explore any barriers that PhD students encounter whilst completing the degree, and develop recommendations for consideration in formulating support strategies to encourage completion for nurses and midwives enrolled on a PhD degree.	PhD programs within School of Nursing and Midwifery at a single university in Australia.	*Design*: Mixed‐methods study. *Method*: Questionnaire and qualitative analysis of open‐ended questions. *Participants*: 16 participants. Nursing and midwifery doctoral students.	Higher degrees enhanced contribution to research and professional development. High attrition rates is a barrier to PhD completion globally. Supervisors and supervision were the focus of the participant's responses and issues were identified in negotiating the right supervisors for PhD students. Selection of supervisors is largely a random and haphazard process. Too often supervisors were changed (without it being requested by the student) PhD students desired more specific guidance from supervisors Supervisors' feedback was often inadequate.	Matching skills and knowledge and understanding is crucial for clinical impact. Academia should refine strategies to better connect clinical practice with PhD education. Embedding structured teaching in supervision can enhance PhD completion rates. Students need explicit instruction on research skills, not just broad feedback Fostering mentorship can empower students and support their academic progression.
Havenga and Sengane (2018), *Challenges experienced by postgraduate nursing students at a South African university*. South Africa	To explore and describe the challenges experienced by postgraduate nursing students enrolled in postgraduate coursework and research programmes at a South African university.	South African university nursing program. Postgraduate nursing students, doctoral, master's and honours students.	*Design*: Qualitative study. *Methods*: Self‐reporting narratives. *Participants*: 15 participants. Postgraduate nursing students' coursework and research programmes.	Students faced difficulties with information literacy and supervision. Limited computer literacy and difficulty assessing academic resources. Delayed supervisor allocation and unstructured feedback. Some supervisors were perceived as lacking passion, being unapproachable and rigid.	Information literacy is required in postgraduate programmes and in nursing. However, many nurses who currently enrol in postgraduate studies are of an older generation that requires additional assistance in terms of information literacy. They should be able to use the newest research evidence in their daily practice and therefore, developing postgraduate students' information literacy as an essential part of postgraduate education. The supervisor–supervisee relationship is central to progress and completion.
Molassiotis et al. (2020), *Doctoral nursing education in east and Southeast Asia: characteristics of the programs and students' experiences of and satisfaction with their studies*. China	To describe the characteristics of nursing doctoral programs in East and Southeast Asian (ESEA) countries and regions from the views of doctoral program coordinators and to explore the students' experiences of and satisfaction with their doctoral nursing program.	East and Southeast Asian nursing PhD programs.	*Design*: Quantitative study. *Method*: Cross‐sectional questionnaires. *Participants*: 193 participants. Nursing doctoral students.	Issues included lack of quality assurance (29.2%), faculty educational background (12.5%), insufficient PhD supervisors (16.7%), and limited time for research (8.3%). Eight of 10 students reported feeling pressured by supervisors to publish research papers. Less than 70% of students felt their institutions promoted cultural diversity and staff‐student interactions.	Increasing the faculty‐to‐student ratio was emphasised to improve supervision quality.
Raffing et al. (2017), *Self‐reported needs for improving the supervision competence of PhD supervisors from the medical sciences in Denmark*. Denmark	To explore PhD supervisors' self‐reported needs and wishes regarding the content of a new program in supervision, with a special focus on the supervision of PhD students in medical fields.	Danish clinical PhD programs. Health sciences.	*Design*: Qualitative study. *Method*: Interviews. *Participants*: 20 participants. PhD supervisors.	Challenges ranged from psycho‐social issues, miscommunication, recruitment difficulties, conflicts involving students and/or (other) supervisors and disruptions Patient care takes priority, affecting supervision dynamics. Supervisors seek training specific to clinical PhD supervision. Clinical supervisors struggle to manage both patient care and student supervision. Many students, especially those with clinical backgrounds, lacked adequate preparation for academic writing and research. Slow recruitment in clinical research impacts PhD timelines and deadlines. Supervisors reported that guiding students through the process of writing their first scientific article was particularly time‐consuming. Supervisors need clearer guidelines to ensure consistent supervision quality.	Establish comprehensive guidelines and role definitions to clarify supervisors' responsibilities and expectations. Develop structured training programs for PhD supervisors that include topics such as managing supervisory relationships, effective communication and psychosocial support for students. These programmes should also address discipline‐specific needs and the unique demands of clinical settings. Address challenges like scheduling in busy environments, guiding first‐time writers and managing patient recruitment delays. Equip supervisors to mentor students on writing their first article while strengthening students' academic writing skills.
Taylor et al. (2018), *Desirable qualities of modern doctorate advisors in the USA: a view through the lenses of candidates, graduates and academic advisors*. USA	To explore desirable qualities for modern doctorate academic advisors in the USA that provide for successful completion were explored.	U.S. doctoral education. Health sciences and business.	*Design*: Mixed‐method study. *Method*: Questionnaire, interviews and open‐ended questionnaire responses. *Participants*: 31 participated in interviews and 189 in questionnaires. Academic advisors and doctoral candidates/recent graduates.	Candidates felt a lack of shared passion and expertise with supervisors. Doctoral research often did not align with advisors' specific expertise. Advisors emphasised maintaining rigorous standards in writing, research and methodology. Advisors highlighted the importance of dedication to quality supervision. Candidates valued clear communication, structured timelines and supportive guidance. Strong advising relationships were built on trust and open communication. Honest dialogue and relationship of trust were the two highest ranked ways of advising by candidates and recent graduates.	Reflective Advising—Advisors should assess their feedback and communication approaches to build trust. Strong relationships and tailored resources enhance supervision quality. A focus on consistent communication, relationships based around honest feedback and personal connection and resources that align with the needs of the candidates are areas that can strengthen the supervision in doctoral programs.
Volkert et al. (2018), *Student motivation, stressors and intent to leave nursing doctoral study: A national study using path analysis*. USA	To examine how the effects of environmental stressors predict the students' intent to leave their current program of doctoral study.	U.S. study of Ph.D. and DNP students in nursing.	*Design*: Quantitative study. *Method*: Questionnaire. *Participants*: 835 Ph.D. and DNP participants.	Stressors related to program issues, primarily relationships between student and faculty/advisor, significantly predict intent to leave.	Nursing faculty must be equipped to mentor and role‐model for DNP and PhD students. Faculty should acknowledge and navigate power differences in student‐adviser relationships. Designating faculty as mentors can provide consistent support, but workload adjustments are essential.
Wieck et al. (2014), *Using a strengths model to build and on‐line nursing education program*. USA	To describe how a faculty used a strengths model to design and build an on‐line nursing doctoral education program.	Online doctoral nursing program with American students living in Japan, Mongolia and Indonesia. Nursing PhD program.	*Method*: Case study of program implementation. *Participants*: 83 participants. PhD students.	Faculty faced challenges in maintaining meaningful relationships with students at a distance. The lack of face‐to‐face interaction made it difficult to provide personalised mentorship and support. Students entered the program with varying levels of preparedness and strengths, which required faculty to adopt highly individualised approaches to support their academic success. Balancing work, family and academic responsibilities was particularly challenging for non‐traditional students. Students from diverse cultural and generational backgrounds had different learning expectations and needs, requiring faculty to adapt teaching and mentoring styles accordingly.	Focus on students' inherent capabilities rather than their deficits. Integrates students in their own communities which are viewed as a base of opportunity. The academic enterprise, including the student and faculty team, becomes a partner in measuring and testing the community to strengthen the nursing mission. If the goal is to have a workforce that mirrors the population, academia must make advanced education available and relevant to the communities. Using a strengths‐based approach to an on‐line education program expands opportunity for all. Adapts learning environments to fit the expectations of tech‐savvy nurses. Implement deliberate strategies like the “PhrnDz Café” (Friends Café) to foster connection in virtual learning.

The results to the two research questions are presented below, by presenting the identified themes: Communication Issues, Structural and Institutional Barriers, Student Preparedness and Adaptation, Establishing Clear Expectations and Feedback to Address Misaligned Roles and Communication Gaps, Institutional Support to Mitigate Structural and Resource‐Related Barriers, Student Empowerment and Training to Support Adaptation and Ethical Awareness.

### 3.2 Factors That Contribute to Ethical Challenges in Doctoral Supervision

#### 3.2.1 Communication Issues

A recurring issue identified across studies was the misalignment of expectations between supervisors and doctoral students. Supervisors often overestimated the adequacy of their support, while students felt that their needs for guidance and emotional support remained unmet (Anttila et al. 2024; Raffing et al. 2017; Geraghty and Oliver 2018; Alves et al. 2023). This disconnect frequently led to dissatisfaction, frustration and hindered academic progress. Additionally, the emphasis supervisors placed on publishing research papers further contributed to students' stress and anxiety (Alves et al. 2023; Molassiotis et al. 2020).

The quality of feedback emerged as a critical concern. Students described feedback as vague or lacking actionable guidance, which created uncertainty about their academic progress (Taylor et al. 2018; Geraghty and Oliver 2018; Molassiotis et al. 2020). Inadequate or unclear feedback was often also cited as an important ethical issue, particularly when it hindered students' ability to succeed (Anttila et al. 2024; Geraghty and Oliver 2018; Alves et al. 2023; Havenga and Sengane 2018). While supervisors emphasised the reciprocal nature of informational support and placed responsibility on the candidate, this perspective was not shared by the doctoral students (Anttila et al. 2024).

Furthermore, students highlighted the need for open and honest dialogue to build trust and foster a productive supervisory relationship (Taylor et al. 2018). However, some perceived their supervisors as unapproachable, rigid or disengaged from the supervisory process, which further exacerbated communication barriers (Havenga and Sengane 2018).

#### 3.2.2 Structural and Institutional Barriers

While not ethical issues in themselves, structural limitations, such as low faculty‐to‐student ratios and inadequate supervisory resources, negatively affected the quality of supervision. These constraints led to delayed feedback and reduced availability of supervisors, limiting their ability to provide meaningful support (Raffing et al. 2017; Molassiotis et al. 2020).

Supervisors working in clinical settings faced additional challenges in balancing patient care and academic responsibilities (the former often being prioritised to the detriment of the latter), often resulting in inconsistent meeting schedules and untimely feedback (Raffing et al. 2017). Furthermore, random or poorly structured student‐supervisor matching processes contributed to unsuccessful supervisory relationships (Geraghty and Oliver 2018). Supervisors expressed a strong need for competence enhancement programmes, particularly in clinical contexts (Raffing et al. 2017). These contextual factors were identified as limiting supervisors' ability to engage consistently in supervisory activities.

#### 3.2.3 Student Preparedness and Adaptation

The transition from clinical practice to academia posed challenges for doctoral students, many of whom felt unprepared for academic tasks such as writing research proposals, applying for grants and scholarly publishing (Fang and Bednash 2017; Fang et al. 2016; Wieck et al. 2014; Raffing et al. 2017). While these challenges may initially appear as personal shortcomings, they were frequently identified as shaped or exacerbated by insufficient orientation, unclear expectations or inadequate communication from supervisors and institutions (Alves et al. 2023). This lack of preparedness increased stress levels and slowed academic progress. Supervisors noted that guiding students through their first scientific article was particularly time‐consuming, caused by students not being properly prepared for academia (Raffing et al. 2017).

Students also faced psychological and emotional challenges, including limited autonomy, time management difficulties, work‐life imbalance, isolation and pressure to publish (Molassiotis et al. 2020). While these stressors frequently hindered well‐being and academic progress, in some cases they also served as motivation for growth. For example, frustration could lead to improvements in research papers or relief following successful manuscript acceptance (Alves et al. 2023).

Low attrition rates were another major concern, reported in multiple studies (Volkert et al. 2018; Geraghty and Oliver 2018). Additionally, cultural and generational differences between students and supervisors often led to misunderstandings and mismatches in mentoring styles, further complicating students' adaptation to academic environments (Wieck et al. 2014; Anttila et al. 2024; Molassiotis et al. 2020). These findings suggest that adaptation difficulties should not be interpreted solely as personal insufficiencies but should be understood within the broader relational and institutional context of doctoral supervision.

To better map our understanding of the origins or *locus* of these ethical challenges, we suggest it can be helpful to classify them across three levels (Table 3): (Breslin et al. 2015) individual‐level factors, including lack of preparedness, miscommunication or supervisory misconduct; (Joseph et al. 2021) institutional‐level factors, such as high student‐supervisor ratios, lack of training or insufficient policy guidance; and (WHO 2021) cultural‐level factors, including academic hierarchies, generational gaps or regional norms around autonomy and deference. Direct ethical issues—such as conflicts of interest, abuse of power and neglect of duty—often emerged at the individual level but were frequently shaped or exacerbated by institutional and cultural conditions. This multi‐level classification clarifies how indirect structural or cultural factors may create conditions under which ethical breaches are more likely to occur.

**TABLE 3:** Classification of ethical challenges in doctoral supervision.

Level	Examples of causes	Type of ethical challenge	Examples from included studies
Individual	Poor communication, lack of preparedness, supervisor misconduct	Direct (e.g., breach of duty)	Vague feedback (Alves et al. 2023), power misuse (Volkert et al. 2018)
Institutional	High student‐supervisor ratios, lack of training, unclear supervision policies	Indirect (enabling ethical tensions)	Resource constraints (Havenga and Sengane 2018), poor matching (Geraghty and Oliver 2018)
Cultural	Academic hierarchies, publication pressure, generational and cross‐cultural differences	Indirect (contextual influences)	Publication pressure (Molassiotis et al. 2020), cultural and generational expectations (Wieck et al. 2014)

### 3.3 Strategies to Address the Challenges in Doctoral Supervision

#### 3.3.1 Establishing Clear Expectations and Feedback to Address Misaligned Roles and Communication Gaps

A widely recommended strategy for reducing misalignment between supervisors and students is the establishment of clear expectations at the beginning of the supervisory relationship. Several studies suggest using formal tools such as written supervision agreements, expectation‐setting checklists and orientation sessions to structure the supervision and ensure mutual understanding (Anttila et al. 2024; Alves et al. 2023).

Regular feedback, such as structured check‐ins, provided students with greater clarity about their progress and created opportunities to address challenges proactively (Anttila et al. 2024; Raffing et al. 2017; Geraghty and Oliver 2018). Additionally, students emphasised the importance of honest dialogue and relationships built on trust as essential components of effective communication (Taylor et al. 2018).

Supervisors, in turn, emphasised the need for clear, written expectations to maintain alignment and uphold academic standards, particularly in academic writing, research methodology and statistical competence (Taylor et al. 2018). To support this, some studies recommended mandatory pre‐service training for students to prepare them for academic roles (Fang and Bednash 2017; Fang et al. 2016).

#### 3.3.2 Institutional Support to Mitigate Structural and Resource‐Related Barriers

A range of institutional support was identified to address structural and resource‐related challenges in doctoral education. Mentorship programmes emerged as particularly effective in reducing student isolation and ensuring consistent academic and emotional guidance. These programmes foster collaboration and strengthen relationships between students and faculty, ultimately improving the overall doctoral experience (Volkert et al. 2018; Wieck et al. 2014).

Supervisor training initiatives were another key recommendation. These programmes aim to equip supervisors with practical skills to manage diverse student needs, especially in clinical or resource‐constrained contexts. Key components included training in effective communication, psychosocial support and supervision under challenging conditions (Anttila et al. 2024; Raffing et al. 2017). Importantly, to address ethical issues such as power imbalances and misaligned priorities, training modules also emphasised research integrity, role boundaries and principles of student‐centred supervision (Volkert et al. 2018; Fang and Bednash 2017; Anttila et al. 2024; Raffing et al. 2017). To further reduce mismatched expectations, some programmes reportedly implemented admission agreements that clearly outline program requirements. One such approach included a mandatory 3‐day orientation, during which students complete a strengths assessment (e.g., StrengthsFinder 2.0) to facilitate expectation alignment and personal development planning (Wieck et al. 2014). In addition, flexible teaching and delivery methods—such as satellite campuses, evening and weekend sessions—were highlighted as crucial for enhancing access and engagement, particularly in under‐resourced or geographically dispersed settings (Havenga and Sengane 2018). Finally, the use of structured supervisory frameworks—including clear role definitions, expectations and standardised guidelines—helped improve consistency across programmes and enhanced the quality of supervision (Raffing et al. 2017).

#### 3.3.3 Student Empowerment and Training to Support Adaptation and Ethical Awareness

To address gaps in students' academic and research skills, targeted training programs were recommended. These initiatives supported students in building confidence in key areas such as grant writing, academic publishing and research methodology. Strengthening these competencies not only reduced stress but also enhanced students' ability to meet the demands of doctoral studies (Fang and Bednash 2017; Fang et al. 2016; Alves et al. 2023). In addition to academic preparation, several studies highlighted the importance of fostering students' ethical awareness and self‐advocacy skills (Geraghty and Oliver 2018; Wieck et al. 2014). Also, mentorship was suggested to empower students and support them in academia (Geraghty and Oliver 2018). Training modules on research integrity, supervisory rights and responsibilities and ethical dilemmas in academia can empower students to recognise and respond to inappropriate supervisory behaviour (Raffing et al. 2017).

Personalised support tailored to individual strengths fosters a more inclusive and supportive learning experience (Wieck et al. 2014; Raffing et al. 2017). Online education programmes that adopt strengths‐based approaches prove particularly effective, allowing students to leverage their unique capabilities and succeed in diverse academic environments (Wieck et al. 2014).

Moreover, universities were encouraged to improve access to support structures to provide students with safe, confidential avenues to seek advice or report concerns (Volkert et al. 2018; Alves et al. 2023).

## 4 Discussion

This review highlights the ethical challenges in doctoral supervision within nursing and health sciences. These challenges are shaped by the student‐supervisor relationship and influenced by communication barriers, institutional constraints and the transition from clinical practice to academia. In this study, we want to distinguish core ethical challenges—such as breaches of trust and power imbalances—from contextual factors like unclear policies or high student–teacher ratios. Using a multi‐level framework (individual, institutional, cultural), we show that ethical supervision requires both structural reform and ethical leadership (van den Akker et al. 2009). Drawing on the concept of ethical leadership—as appropriate conduct demonstrated through action, communication and decisions (Brown et al. 2005)—we emphasise the shared responsibility of supervisors and doctoral students. Supervisors act as moral managers by modelling fairness and integrity, while students contribute through active ethical engagement and personal responsibility. Notably, few studies addressed strategies that support or develop students' ethical agency, such as training in ethical awareness or access to third‐party support channels. This represents an important gap, as students' ability to recognise and respond to inappropriate supervision practices is crucial to mitigating ethical risks.

A recurring issue is students' stress and dissatisfaction, often stemming from misaligned expectations. Unclear roles and responsibilities can lead to academic pressure, overwhelming deadlines and high‐pressure evaluations. These pressures frequently contribute to mental health struggles, including fatigue and sleep deprivation (Schmidt and Hansson 2018; Evans et al. 2018; Jackson et al. 2021c). Given that well‐being is a core value in healthcare, institutions teaching in the field have both an academic and ethical duty to support doctoral students' well‐being (Schmidt and Hansson 2018).

This review aligns with research, such as Castelló et al., who found that nearly one‐third of doctoral students—particularly younger, female and part‐time students—considered dropping out due to difficulties balancing academic, personal and professional responsibilities (Castelló et al. 2017). Without adequate support, these pressures can lead to extended study durations, increased dropout rates and financial burdens for universities (Cornér et al. 2017). Therefore, institutions must implement strategies to promote not only academic success but also holistic well‐being, enabling students to thrive both personally and professionally (Schmidt and Hansson 2018). Ethical challenges may differ between research‐focused (e.g., PhD) and practice‐oriented (e.g., DNP) doctoral programs. While PhD programs emphasise academic research, DNP programs focus more on clinical leadership and evidence‐based practice, potentially leading to dual ethical pressures. However, many included studies did not specify the type of doctoral program, and the PhD/DNP distinction is not used in all countries. These factors limit the ability to draw program‐specific conclusions and should be considered when interpreting the findings.

Supervisors also faced ethical dilemmas, often shaped by structural constraints. A key challenge was balancing multiple roles while supporting diverse student needs. Doctoral students transitioning from clinical practice to academia struggled with research expectations, complicating and weakening the supervisory relationships. Ethical tensions intensify when supervisors act as mentors, evaluators and research collaborators—creating potential conflicts of interest and limiting open dialogue. Similar issues in clinical education show how dual roles can undermine trust (Taherian and Shekarchian 2008), underscoring the need for role clarity and ethical safeguards in supervision. Effective supervision depends on assessing students' skills and competencies, such as knowledge, creativity and communication (Friedrich‐Nel and Mac Kinnon 2019). Differences in expectations and mismatched supervision styles can create challenges (Evans and Stevenson 2011); however, which unfortunately might not be met as academia often lacks the needed open forums for discussing such challenges. This reportedly leaves supervisors feeling isolated (Shaw 2014). As communication and relationship‐building require effort from both supervisors and students, such difficulties are felt by both parties.

Our findings also indicate that the nature and prioritisation of ethical challenges vary by regional and economic context. In lower‐ and middle‐income countries (e.g., South Africa), prominent concerns included limited information literacy, restricted access to qualified supervisors and insufficient institutional support (Geraghty and Oliver 2018). These issues often reflect broader structural barriers, such as under‐resourced universities and high student‐to‐supervisor ratios. In contrast, studies from high‐income regions (e.g., East Asia and Scandinavia) focused more on pressures to publish, student autonomy and tensions between academic freedom and supervisory authority (Taylor et al. 2018; Alves et al. 2023). However, it is important to note that most included studies originated from high‐income countries, with only one from a low‐income setting. This geographic concentration limits the ability to capture the full range of ethical challenges globally, particularly in underrepresented regions where resource disparities may shape distinct risks. While some studies mentioned cultural norms, such as autonomy and academic authority, the review lacked in‐depth cross‐cultural analysis. Ethical tensions linked to collectivist values, such as the conflict between student obedience and academic independence, were largely underexplored. Recent research confirms that socio‐cultural factors significantly influence doctoral students' ethical experiences and supervisory relationships, yet they remain insufficiently examined across diverse contexts (Thao and Trut Thuy 2024).

In some countries, doctoral students are evaluated based on their undergraduate performance to assess their potential for success (Friedrich‐Nel and Mac Kinnon 2019). As the gap from undergraduate to graduate studies is perceived to be quite large by many doctoral students, this evaluation might be a poor guide of future achievements, leading to difficulties with the matching process. This process goes both ways (Pizzolato and Dierickx 2023). Supervisors may also assume that students are well‐versed in research integrity, yet competitive research environments can lead to ethical lapses. This highlights the relevance of the established ethics principles: beneficence, non‐maleficence, autonomy and justice.

The principles of biomedical ethics provide a valuable framework for addressing ethical challenges in doctoral supervision (Beauchamp and Childress 2019). Beneficence, in this context, highlights the supervisor's responsibility to actively support students' academic and personal development, fostering a positive and constructive learning environment (van Rooij et al. 2021). A successful PhD also produces important knowledge that will benefit patients and other stakeholders. At the same time, non‐maleficence—the obligation to “do no harm”—requires supervisors to recognise and mitigate the psychological and emotional toll of doctoral studies (Löfström and Pyhältö 2017). Excessive academic pressure, unclear expectations or neglect of students' well‐being can lead to distress and scientific failure too, making it essential for supervisors to remain attentive and proactive in supporting student well‐being (van Rooij et al. 2021).

Equally important is autonomy, which ensures that doctoral students have the space to make academic decisions, express ideas and shape their research. While some students develop their own research plans, others work within predefined projects; however, even in the latter case, their perspectives and interests should be acknowledged (Löfström et al. 2023). Transparent communication and adequate support structures are essential to balancing institutional expectations with students' academic independence (van Rooij et al. 2021).

Finally, justice calls for fairness in supervision, ensuring that students are not arbitrarily assigned to supervisors but matched based on compatibility and research interests. Students facing significant academic or personal challenges should receive tailored support to ensure equitable access to supervision and resources. For instance, clinicians transitioning into academia often find themselves in a novice role again and may benefit from structured guidance to bridge the gap between practice and research. Developing targeted strategies to integrate clinical experience with scientific inquiry can enhance program completion rates and prepare nurse scientists to advance disciplinary knowledge (Armstrong et al. 2017). Nevertheless, supervisors are often expected to be the primary source of support for the doctoral student (Byrom et al. 2022). While supervisors and institutions hold primary responsibility for ethical supervision, students also require support in developing ethical agency. This includes knowing their rights, recognising problematic supervisory dynamics and knowing when and how to seek third‐party support. Structured training in academic ethics, peer mentoring networks and formalised complaint mechanisms can help build student capacity. Such measures foster shared responsibility and contribute to a more transparent and resilient doctoral environment (Williams et al. 2021). By integrating these ethical principles into supervisory practices, universities can foster a more supportive, fair and transparent doctoral environment, ultimately improving both student well‐being and supervisors' ability to successfully guide students to accomplish their degrees.

Last, this predominant focus on the doctoral student perspective in the included studies means that there is limited attention to the ethical dilemmas faced by supervisors in the material. Issues such as role conflict, balancing clinical and academic responsibilities and navigating dual roles remain underexplored. This underrepresentation may partly reflect a broader trend: PhD nursing programs in the United States have seen a 17.5% decline in enrolment since 2013 (American Association of Colleges of Nursing 2024), leading to fewer nurse supervisors and, consequently, fewer studies capturing their perspectives.

### 4.1 Strengths and Limitations

To minimise bias, the review was supported by a medical librarian at the Uppsala University who guided database selection and tailored search strategy. Despite this effort, some limitations should be acknowledged. A major challenge was the limited availability of primary research explicitly addressing ethical challenges in doctoral supervision. This may reflect both inadequate indexing and the broader lack of attention to ethics in doctoral education, as noted in a previous systematic review (Haley et al. 2025). While our broad search terms were designed to capture a wide range of ethical concerns, it is possible that studies addressing latent or implicit issues (e.g., power imbalances or supervisory tensions) using non‐ethical language were missed. Additionally, extracting information was sometimes complex, as certain challenges—such as ineffective communication between supervisors and students—could be interpreted as ethical issues and/or as structural or linguistic problems.

We limited our review to peer‐reviewed literature indexed in Scopus and Web of Science, published from 2014 onwards. This approach may have excluded earlier foundational studies and grey literature, such as dissertations or industry reports, which could offer valuable practical insights. Future reviews could expand the timeframe and include grey literature to enhance contextual depth.

Moreover, most included studies did not clearly distinguish between research‐oriented (PhD) and practice‐oriented (DNP) programs. This limited our ability to examine potential differences in ethical challenges, such as those related to the dual clinical and academic responsibilities often present in DNP programs.

While our inclusion criteria limited the review to studies published in English, it is important to acknowledge that region‐specific ethical challenges, particularly in non‐English‐speaking and resource‐constrained settings such as parts of Asia and Latin America, may differ in important ways.

The included studies were predominantly from high‐income countries, with only one from a low‐income setting, which limits the global relevance of our findings. Ethical challenges in doctoral supervision may manifest differently across contexts, and issues such as limited supervision capacity or institutional support may be underrepresented in this synthesis.

Further, the relatively small number of included studies may limit the generalisability. For example, the impact of cultural differences on supervision ethics was addressed in few studies, making it difficult to draw robust cross‐cultural comparisons. While most qualitative research focused on doctoral students' experiences, few offered in‐depth insight from supervisors. This may have limited the analysis of supervisor‐specific ethical dilemmas. While many of the qualitative studies had small sample sizes, this is typical and appropriate for in‐depth exploration of complex ethical issues. As our aim was thematic synthesis, statistical power was not a relevant consideration.

Although the included studies were generally methodologically sound, some qualitative studies lacked full reporting of COREQ elements, such as interviewer experience and training. This is consistent with findings from previous reviews, which show that such details are frequently underreported, even in high‐impact journals. For example, only 37% of qualitative health research articles reported interviewer credentials, and just 14% described the interviewers' prior experience or training (O'Brien et al. 2014). Further, several COREQ criteria were not applicable for the mixed‐methods studies, where data were collected through open‐ended survey questions rather than interviews. While our appraisal process involved independent dual reviewers and consensus meetings to ensure rigour, the thematic synthesis may still reflect variability in study quality.

### 4.2 Suggestions for Academic Practice

- At the beginning of the supervisory relationship, clearly define roles, responsibilities and feedback timelines. This helps establish trust and mutual understanding, fostering a productive and supportive environment.
- Schedule structured check‐ins to discuss progress and address challenges proactively. Ensure that feedback is timely, specific and constructive to support students navigating their academic journey effectively.
- Implement mentorship programmes and supervisor training initiatives to reduce student isolation and ensure consistent guidance. Provide resources that support emotional and psychological well‐being, such as peer support networks and mental health services, to foster resilience and sustained academic engagement.

### 4.3 Suggestions for Future Research

- Conduct in‐depth studies on the ethical challenges faced by both supervisors and doctoral students in nursing, focusing on issues such as power dynamics, conflicts of interest and the impact of cultural and generational differences. This research could provide valuable insights into how these dilemmas affect the supervisory relationship and suggest strategies for fostering ethical and supportive interactions. Future research should consider incorporating studies from regions underrepresented in this study, possibly through multilingual reviews, to capture a more comprehensive and globally representative understanding of ethical challenges in supervision.
- Investigate the impact of comprehensive supervisor training initiatives on ethical supervision practices. Further, assess how these programmes influence supervisors' ability to handle ethical issues, provide constructive feedback and support students' academic and personal development. This research could help identify best practices and areas for improvement in supervisor training.

## 5 Conclusions

Ethical supervision is foundational to the success of doctoral education in nursing and health sciences, particularly as students navigate the complexities of academic and clinical environments. Global nursing education needs to integrate ethics further into doctoral training. This review highlights the need for institutions to move beyond reactive solutions and to embed proactive, ethically grounded supervision policies. Practical implications include the adoption of supervision agreements, structured feedback systems and mandatory training modules to help both supervisors and students to recognise and address ethical tensions. From a policy perspective, academic institutions should ensure oversight mechanisms—such as supervision audits or peer review of supervisory practices—are established to uphold transparency and accountability. Resources that support students' emotional well‐being should be institutionalised rather than optional. Further research is needed to evaluate the effectiveness of these interventions across diverse cultural, institutional and programmatic contexts and to explore supervisors' ethical experiences more deeply.

## Author Contributions

**Tove Godskesen:** conceptualisation, investigation, methodology, validation, formal analysis, writing – original draft, project administration, writing – review and editing. **Maria Grandahl:** methodology, formal analysis (quality evaluation), writing – review and editing. **Amalie Nilsen Hagen:** methodology, formal analysis (quality evaluation), writing – review and editing. **Stefan Eriksson:** conceptualisation, investigation, methodology, validation, formal analysis, writing – original draft, writing – review and editing.

## Disclosure

Protocol: No protocol was developed for this study.

## Conflicts of Interest

The authors declare no conflicts of interest. All authors are involved in doctoral supervision and/or supervision training programmes within their respective institutions. While this professional experience informed the research focus, it did not influence the data collection (performed with an external university librarian), analysis or interpretation. The authors upheld independence through a structured and transparent review process, in line with PRISMA guidelines. We confirm that we have followed the journal's author guidelines.

## Supporting information


**Appendix S1:** jan70298‐sup‐0001‐AppendixS1.docx.

**Appendix S2:** jan70298‐sup‐0002‐AppendixS2.docx.

**Appendix S3:** jan70298‐sup‐0003‐AppendixS3.docx.

## Data Availability

All data are presented in the article.
