# Supplementary material for: Ethical Challenges and Strategies in Nursing Doctoral Supervision: A Systematic Mixed‐Method Review
Source: J Adv Nurs. 2025 Oct 23;82(5):4167–84. doi: 10.1111/jan.70298 (PMC13069218; doi:10.1111/jan.70298)
Supplement: Supplementary file 2 — Appendix S2: jan70298‐sup‐0002‐AppendixS2.docx. [file JAN-82-4167-s002.docx]

**Cinahl 24-12-06** **(initial search) + 25-09-15 (updated search)**

| Söknummer | Söktermer | Antal referenser |
| --- | --- | --- |
| 1 | "Ethics+" OR "Morals+" OR  "Social Values"  MH  ethic* OR moral* OR value* OR "research integrity" OR respons* OR accountab*  TI/AB | 1,097,329  +  1,137,177 |
| 2 | "Mentorship" OR "Faculty-Student Relations" OR "Supervisors and Supervision+" OR "Interpersonal Relations"  MH  supervis* OR mentor***** OR advisor***** OR advice* OR "academic advising" OR advisement* OR "research leadership" OR guidance OR  strateg* OR framework*  TI/AB | 300,083  +  320,094 |
| 3 | "Education, Doctoral" OR  "Education, Nursing, Doctoral" OR "Doctorally Prepared Nurses"  MH  "doctoral degree*" OR "doctoral dissertation*" OR "doctoral program*" OR "doctoral nursing program*" OR "doctoral stud*" OR PhD  TI/AB | 10,642  +  10,936 |
| 4 | "Nurses+"  MH  nurs* OR "health care science" OR  "health science*" OR "medical science*" OR "caring science*" OR "nursing science*" OR "health research" OR "medical research" OR "caring research" OR "nursing research" OR "public health"  TI/AB | 798,150  +  827,476 |
| 5 | 1 AND 2 AND 3 AND 4 | 171 + 181 |
| 6 | 5 AND Limiters - Publication Date: 20140101- | 113 + 18 |

**Education Source 24-12-06 (initial search) + 25-09-15 (updated search)**

| Söknummer | Söktermer | Antal referenser |
| --- | --- | --- |
| 1 | ethic* OR moral* OR value* OR "research integrity" OR respons* OR accountab*  TI/AB/SU | 659,498  +  701,346 |
| 2 | supervis* OR mentor***** OR advisor***** OR advice* OR "academic advising" OR advisement* OR "research leadership" OR guidance OR  strateg* OR framework*    TI/AB/SU | 572,502  +  290,263 |
| 3 | "doctoral degree*" OR "doctoral dissertation*" OR "doctoral program*" OR "doctoral nursing program*" OR "doctoral stud*" OR PhD  TI/AB/SU | 21,604  +  23,151 |
| 4 | nurs* OR "health care science" OR  "health science*" OR "medical science*" OR "caring science*" OR "nursing science*" OR "health research" OR "medical research" OR "caring research" OR "nursing research" OR "public health"  TI/AB/SU | 119,888  +  122,658 |
| 5 | 1 AND 2 AND 3 AND 4 | 112 + 81 |
| 6 | 5 AND Limiters - Published Date: 20140101- | 90 + 11 |

**ERIC 24-12-06 (initial search) + 25-09-15 (updated search)**

| Söknummer | Söktermer | Antal referenser |
| --- | --- | --- |
| 1 | ethic* OR moral* OR value* OR "research integrity" OR respons* OR accountab*  TI/AB/SU | 460,380  +  475,723 |
| 2 | supervis* OR mentor***** OR advisor***** OR advice* OR "academic advising" OR advisement* OR "research leadership" OR guidance OR  strateg* OR framework*    TI/AB/SU | 193,002  +  202,375 |
| 3 | "doctoral degree*" OR "doctoral dissertation*" OR "doctoral program*" OR "doctoral nursing program*" OR "doctoral stud*" OR PhD  TI/AB/SU | 21,191  +  21,962 |
| 4 | nurs* OR "health care science" OR  "health science*" OR "medical science*" OR "caring science*" OR "nursing science*" OR "health research" OR "medical research" OR "caring research" OR "nursing research" OR "public health"  TI/AB/SU | 31,207  +  31,990 |
| 5 | 1 AND 2 AND 3 AND 4 | 30 + 31 |
| 6 | 5 AND Limiters - Published Date: 20140101- | 13 + 1 |

**PubMed 24-12-06 (initial search) + 25-09-15 (updated search)**

| Söknummer | Söktermer | Antal referenser |
| --- | --- | --- |
| 1 | "Ethics"[Mesh] OR "Morals"[Mesh] OR "Social Values"[Mesh]  OR  ethic* OR moral* OR value* OR "research integrity" OR respons* OR accountab*  [Title/Abstract] | 6,641,126  +  6,948,625 |
| 2 | "Mentors"[Mesh] OR "Interpersonal Relations"[Mesh]  OR  supervis* OR mentor***** OR advisor***** OR advice* OR "academic advising" OR advisement* OR "research leadership" OR guidance OR  strateg* OR framework*  [Title/Abstract] | 2,798,968  +  3,065,669 |
| 3 | "doctoral degree*" OR "doctoral dissertation*" OR "doctoral program*" OR "doctoral nursing program*" OR "doctoral stud*" OR PhD  [Title/Abstract] | 13,554  +  14,364 |
| 4 | "Nurses"[Mesh]  OR  nurs* OR "health care science" OR  "health science*" OR "medical science*" OR "caring science*" OR "nursing science*" OR "health research" OR "medical research" OR "caring research" OR "nursing research" OR "public health"  [Title/Abstract] | 1,086,810  +  1,150,054 |
| 5 | 1 AND 2 AND 3 AND 4 | 262 + 286 |
| 6 | 5 AND Limiters - Publication Date: 20140101- | 182 + 46 |

**Scopus 24-12-06 (initial search) + 25-09-15 (updated search**

| Söknummer | Söktermer | Antal referenser |
| --- | --- | --- |
| 1 | ethic* OR moral* OR value* OR "research integrity" OR respons* OR accountab*  Title/Abstract/Keywords | 17,722,224  +  1,137,177 |
| 2 | supervis* OR mentor***** OR advisor***** OR advice* OR "academic advising" OR advisement* OR "research leadership" OR guidance OR  strateg* OR framework*    Title/Abstract/Keywords | 7,993,784  +  320,094 |
| 3 | "doctoral degree*" OR "doctoral dissertation*" OR "doctoral program*" OR "doctoral nursing program*" OR "doctoral stud*" OR PhD  Title/Abstract/Keywords | 51,281  +  10,936 |
| 4 | nurs* OR "health care science" OR  "health science*" OR "medical science*" OR "caring science*" OR "nursing science*" OR "health research" OR "medical research" OR "caring research" OR "nursing research" OR "public health"  Title/Abstract/Keywords | 2,148,156  +  827,476 |
| 5 | 1 AND 2 AND 3 AND 4 | 419 + 181 |
| 6 | 5 AND Limiters - Published Date: 20140101- | 311 + 18 |

**WoS 24-12-06 (initial search) + 25-09-15 (updated search)**

| Söknummer | Söktermer | Antal referenser |
| --- | --- | --- |
| 1 | ethic* OR moral* OR value* OR "research integrity" OR respons* OR accountab*  Title/Abstract/Topic | 12,816,873  +  14,116,593 |
| 2 | supervis* OR mentor***** OR advisor***** OR advice* OR "academic advising" OR advisement* OR "research leadership" OR guidance OR strateg* OR framework*    Title/Abstract/Topic | 6,095,461  +  7,173,915 |
| 3 | "doctoral degree*" OR "doctoral dissertation*" OR "doctoral program*" OR "doctoral nursing program*" OR "doctoral stud*" OR PhD  Title/Abstract/Topic | 34,498  +  41,894 |
| 4 | nurs* OR "health care science" OR "health science*" OR "medical science*" OR "caring science*" OR "nursing science*" OR "health research" OR "medical research" OR "caring research" OR "nursing research" OR "public health"  Title/Abstract/Topic | 954,776  +  1,108,959 |
| 5 | 1 AND 2 AND 3 AND 4 | 237 + 296 |
| 6 | 5 AND Limiters - Published Date: 20140101- | 188 + 57 |
