# Supplementary material for: Ethical Challenges and Strategies in Nursing Doctoral Supervision: A Systematic Mixed‐Method Review
Source: J Adv Nurs. 2025 Oct 23;82(5):4167–84. doi: 10.1111/jan.70298 (PMC13069218; doi:10.1111/jan.70298)
Supplement: Supplementary file 3 — Appendix S3: jan70298‐sup‐0003‐AppendixS3.docx. [file JAN-82-4167-s003.docx]

**Quality appraisal of the included qualitative studies. In mixed-methods studies, COREQ was used for the qualitative data.**

| **COREQ**  **criteria** | **1** | **2** | **3** | **4** | **5** | **6** | **7** | **8** | **9** | **10** | **11** | **12** | **13** | **14** | **15** | **16** | **17** | **18** | **19** | **20** | **21** | **22** | **23** | **24** | **25** | **26** | **27** | **28** | **29** | **30** | **31** | **32** |
| --- | --- | --- | --- | --- | --- | --- | --- | --- | --- | --- | --- | --- | --- | --- | --- | --- | --- | --- | --- | --- | --- | --- | --- | --- | --- | --- | --- | --- | --- | --- | --- | --- |
| Alves et al. (2023) | + | + | + | + | - | - | - | - | + | + | + | + | - | + | - | + | + | - | + | + | + | - | + | - | - | + | + | + | + | + | + | + |
| Anttila et al. (2023) | + | + | + | + | - | - | - | - | + | + | + | + | - | NA | NA | + | NA | NA | NA | NA | NA | NA | NA | + | - | + | - | - | + | + | + | + |
| Geraghty et al. (2018) | NA | - | - | - | - | - | NA | NA | + | + | + | + | - | NA | NA | + | NA | NA | NA | NA | NA | NA | NA | - | + | + | + | - | + | + | + | NA |
| Havenga et al. (2018) | + | - | - | - | - | - | - | - | + | + | + | + | + | NA | NA | - | NA | NA | NA | NA | NA | - | NA | + | - | + | - | - | + | + | + | + |
| Raffing et al. (2017) | - | - | - | - | - | - | - | - | + | + | + | + | - | + | - | + | + | - | + | - | + | - | - | + | - | + | - | - | + | + | + | + |
| Taylor et al.  (2018) | - | - | - | - | - | - | - | - | + | + | + | + | + | - | - | + | + | - | + | + | - | - | - | - | + | + | - | - | + | + | + | + |
| Wieck et al. (2014) | NA | - | + | - | NA | NA | + | NA | + | + | + | + | - | + | NA | - | NA | NA | NA | NA | NA | NA | - | - | - | - | - | - | - | - | + | - |
| **COREQ**  **criteria** | 1 | 2 | 3 | 4 | 5 | 6 | 7 | 8 | 9 | 10 | 11 | 12 | 13 | 14 | 15 | 16 | 17 | 18 | 19 | 20 | 21 | 22 | 23 | 24 | 25 | 26 | 27 | 28 | 29 | 30 | 31 | 32 |

The COREO criteria): (1) Interviewer/facilitator, (2) Credentials, (3) Occupation, (4) Gender, (5) Experience and training, (6) Relationship established, (7) Participant knowledge of the interviewer, (8) Interviewer characteristics, (9) Methodological orientation and theory, (10) Sampling, (11) Method of approach, (12) Sample size, (13) Non-participation, (14) Setting of data collection, (15) Presence of non-participants, (16) Description of sample, (17) Interview guide, (18) Repeat interviews, (19) Audio/visual recording, (20) Field notes, (21) Duration, (22) Data saturation, (23) Transcripts returned, (24) Number of data coders, (25) Description of the coding tree, (26) Derivation of themes, (27) Software, (28) Participant checking, (29) Quotations presented, (30) Data and findings consistent, (31) Clarity of major themes, (32) Clarity of minor themes (Tong et al. 2007)

**Quality appraisal of the included quantitative studies. In mixed-methods studies, STROBE was used for quantitative data.**

| **STROBE criteria** | **1** | **2** | **3** | **4** | **5** | **6** | **7** | **8** | **9** | **10** | **11** | **12** | **13** | **14** | **15** | **16** | **17** | **18** | **19** | **20** | **21** | **22** | **Total score** |
| --- | --- | --- | --- | --- | --- | --- | --- | --- | --- | --- | --- | --- | --- | --- | --- | --- | --- | --- | --- | --- | --- | --- | --- |
| Anttila et al. (2023) | + | + | + | + | + | + | + | + | - | + | + | + | + | + | + | + | - | + | + | + | - | + | 19 |
| Fang et al. (2016) | + | + | + | - | - | + | - | + | + | + | + | + | + | + | + | + | + | - | - | + | - | + | 16 |
| Fang et al. (2017) | + | + | + | + | - | + | - | + | + | + | + | + | + | + | + | + | - | - | - | + | - | + | 16 |
| Geraghty & Oliver (2018) | + | + | + | + | + | + | - | + | - | + | + | + | + | + | + | + | - | + | + | + | + | - | 18 |
| Molassiotis et al. (2020) | + | + | + | + | + | + | + | - | - | - | + | + | - | - | + | + | + | - | + | + | - | + | 15 |
| Taylor et al. (2018) | + | + | + | + | + | - | - | + | - | + | + | + | - | - | + | + | - | + | + | + | + | - | 15 |
| Volkert et al. (2018) | + | + | + | + | + | + | + | - | - | + | - | + | - | + | + | + | + | + | + | + | - | - | 16 |
| **STROBE criteria** | 1 | 2 | 3 | 4 | 5 | 6 | 7 | 8 | 9 | 10 | 11 | 12 | 13 | 14 | 15 | 16 | 17 | 18 | 19 | 20 | 21 | 22 | Total score |

The STROBE criteria: (1) Title and abstract, (2) Background/rationale, (3) Objectives, (4) Study design, (5) Setting, (6) Participants, (7) Variables, (8) data sources/measurement, (9) Bias, (10) Study size, (11) Quantitative variables, (12) Statistical methods, (13) Participants, (14) Descriptive data, (15) Outcome data, (16) Main results , (17) Other analyses, (18) Key results, (19) Limitations, (20) Interpretation, (21) Generalisability, (22) Funding (von Elm et al. 2007)
